# The Physiology of Bone Pain. How Much Do We Really Know?

**DOI:** 10.3389/fphys.2016.00157

**Published:** 2016-04-26

**Authors:** Sara Nencini, Jason J. Ivanusic

**Affiliations:** Department of Anatomy and Neuroscience, University of MelbourneMelbourne, VIC, Australia

**Keywords:** bone pain, pain, nociception, bone, electrophysiology, periosteum, bone marrow

## Abstract

Pain is associated with most bony pathologies. Clinical and experimental observations suggest that bone pain can be derived from noxious stimulation of the periosteum or bone marrow. Sensory neurons are known to innervate the periosteum and marrow cavity, and most of these have a morphology and molecular phenotype consistent with a role in nociception. However, little is known about the physiology of these neurons, and therefore information about mechanisms that generate and maintain bone pain is lacking. The periosteum has received greater attention relative to the bone marrow, reflecting the easier access of the periosteum for experimental assessment. With the electrophysiological preparations used, investigators have been able to record from single periosteal units in isolation, and there is a lot of information available about how they respond to different stimuli, including those that are noxious. In contrast, preparations used to study sensory neurons that innervate the bone marrow have been limited to recording multi-unit activity in whole nerves, and whilst they clearly report responses to noxious stimulation, it is not possible to define responses for single sensory neurons that innervate the bone marrow. There is only limited evidence that peripheral sensory neurons that innervate bone can be sensitized or that they can be activated by multiple stimulus types, and at present this only exists in part for periosteal units. In the central nervous system, it is clear that spinal dorsal horn neurons can be activated by noxious stimuli applied to bone. Some can be sensitized under pathological conditions and may contribute in part to secondary or referred pain associated with bony pathology. Activity related to stimulation of sensory nerves that innervate bone has also been reported in neurons of the spinoparabrachial pathway and the somatosensory cortices, both known for roles in coding information about pain. Whilst these provide some clues as to the way information about bone pain is centrally coded, they need to be expanded to further our understanding of other central territories involved. There is a lot more to learn about the physiology of peripheral sensory neurons that innervate bone and their central projections.

## Introduction

This review aims to summarize and critically evaluate our current understanding of the physiological properties of peripheral sensory neurons that innervate bone, and how information about noxious stimulation coded by these neurons is passed through the central nervous system to the cerebral cortex to elicit painful sensations. We begin by summarizing some key concepts regarding the quality and management of bone pain, and what we know about the morphology and molecular phenotype of bone afferent neurons, and then we explore in detail the physiology of bone afferent neurons and their projections through the CNS.

## Bone pain: clinical and experimental observations

Pain associated with bony pathology, including bone marrow edema syndromes, osteomyelitis, osteoarthritis, fractures, and bone cancer causes a major burden (both in terms of quality of life and cost) on individuals and health care systems worldwide. This burden is expected to increase with advances in modern medicine that prolong life expectancy, because many of the conditions that cause bone pain are intractable and develop late in life. The prevalence of many of these conditions is high, for example, osteoarthritis affects almost 10% of men and 18% of women over 60 years of age (worldwide estimate), and osteoporosis affects up to 30% of postmenopausal women in northern USA (Woolf and Pfleger, 2003). Metastatic bone pain is the most common pain syndrome reported in cancer patients, and up to 50% of patients report the pain being poorly managed by present treatments (Mantyh and Hunt, 2004). Management of bone pain with conventional analgesia is based on the assumption that the mechanisms that mediate bone pain are similar to those that mediate pain in other tissue systems and can therefore be targeted with similar therapies. Opioids and non-steroidal anti-inflammatory drugs (NSAIDs) are generally used to treat mild to severe pain, but therapeutic use for bone pain is limited by undesirable side effects including sedation, respiratory depression, tolerance to prolonged use, risk of addiction, gastrointestinal effects and renal toxicity. All of these occur with prolonged use of the sort required to treat persistent pain in intractable conditions such as osteoarthritis, osteoporosis, and bone cancer. NSAIDs and opioid analgesia use in the treatment of bone pain is further complicated because of the significant undesirable effects on bone remodeling/healing (Bove et al., 2009; Pountos et al., 2012; Chrastil et al., 2013) which complicates the underlying pathology. Other agents that inhibit the activity of osteoclasts (e.g., Osteoprotegerin) or that act by reducing inflammatory processes (e.g., function blocking nerve growth factor antibodies) produce significant analgesia in animal models of bone cancer-induced and fracture pain (Honore et al., 2000a; Halvorson et al., 2005; Sevcik et al., 2005; Jimenez-Andrade et al., 2007; Koewler et al., 2007). These primarily exert effects in the periphery and are targeted at the causes of bone pain. However, they can have significant side effects related to bone remodeling and/or bone destruction that have limited their therapeutic potential (Holmes, 2012; Seidel et al., 2013). There is a clear need to find alternative strategies to treat bone pain that do not involve the use of NSAIDs or opioids, and are targeted more specifically at the neural or inflammatory mechanisms that generate and/or maintain the pain.

The origin of pain associated with bony tissues has been a contentious issue. Early studies noted that direct, noxious mechanical stimulation of the periosteum produced painful percepts in human subjects (Inman and Saunders, 1944), and indeed some more recent literature highlights the prevailing opinion that pain from bone is generally not perceived unless the periosteum is involved (Mach et al., 2002). Pain from periosteum is often described as sharp and well-localized, and occurs for example with fractures significant enough to impact on the periosteum (Santy and Mackintosh, 2001). However, injection of irritants into the medullary cavity is also very painful, as is needle aspiration of bone marrow, and this pain is distinct from that associated with disruption of the periosteum (Niv et al., 2003). In addition, patients often perceive bone pain in pathologies confined principally to the bone marrow that have no obvious periosteal involvement (e.g., intra-osseous engorgement syndrome) (Lemperg and Arnoldi, 1978; Arnoldi, 1990). In these cases, the pain is often described exclusively as dull and diffuse and difficult to localize. Bone cancer-induced pain falls within this latter category, and usually consists of background pain that is described as constant and dull and increases in intensity over time (Honore and Mantyh, 2000; Haegerstam, 2001). In addition, patients with bone cancer often report another more intense pain upon movement or weight-bearing (breakthrough pain) (Portenoy et al., 1999). Thus, it appears that both the periosteum and the marrow cavity of bones must be innervated by primary afferent neurons capable of transducing and transmitting nociceptive information. These bone afferent neurons provide the central nervous system with information that elicits primary pain arising from bone.

Pathology in bone can also produce sensitivity to normally innocuous stimulation (allodynia) and/or increased sensitivity to noxious stimulation (hyperalgesia) of skin around the bone/s involved or even of skin at distant sites. This is often described as secondary or referred pain, and likely reflects sensitization of cutaneous afferent neurons and/or their central projections (Ren and Dubner, 1999a). Sensitization involves increased excitability (reduced stimulus threshold for activation and/or an increased frequency of action potential discharge) of peripheral and central sensory neurons. Many experimental studies reporting pain behavior in animal models of bony pathology use behavioral testing platforms that assay pain, thermal or mechanical sensitivity primarily (or exclusively) at skin around the affected bone (Cain et al., 2001; Urch et al., 2003; Yanagisawa et al., 2010; Uhelski et al., 2013), and so are likely to monitor mechanisms associated with secondary or referred pain, not primary pain associated with direct stimulation of nociceptors in bone.

## Morphology and molecular phenotype of sensory neurons that innervate bone

**Figure 1:**
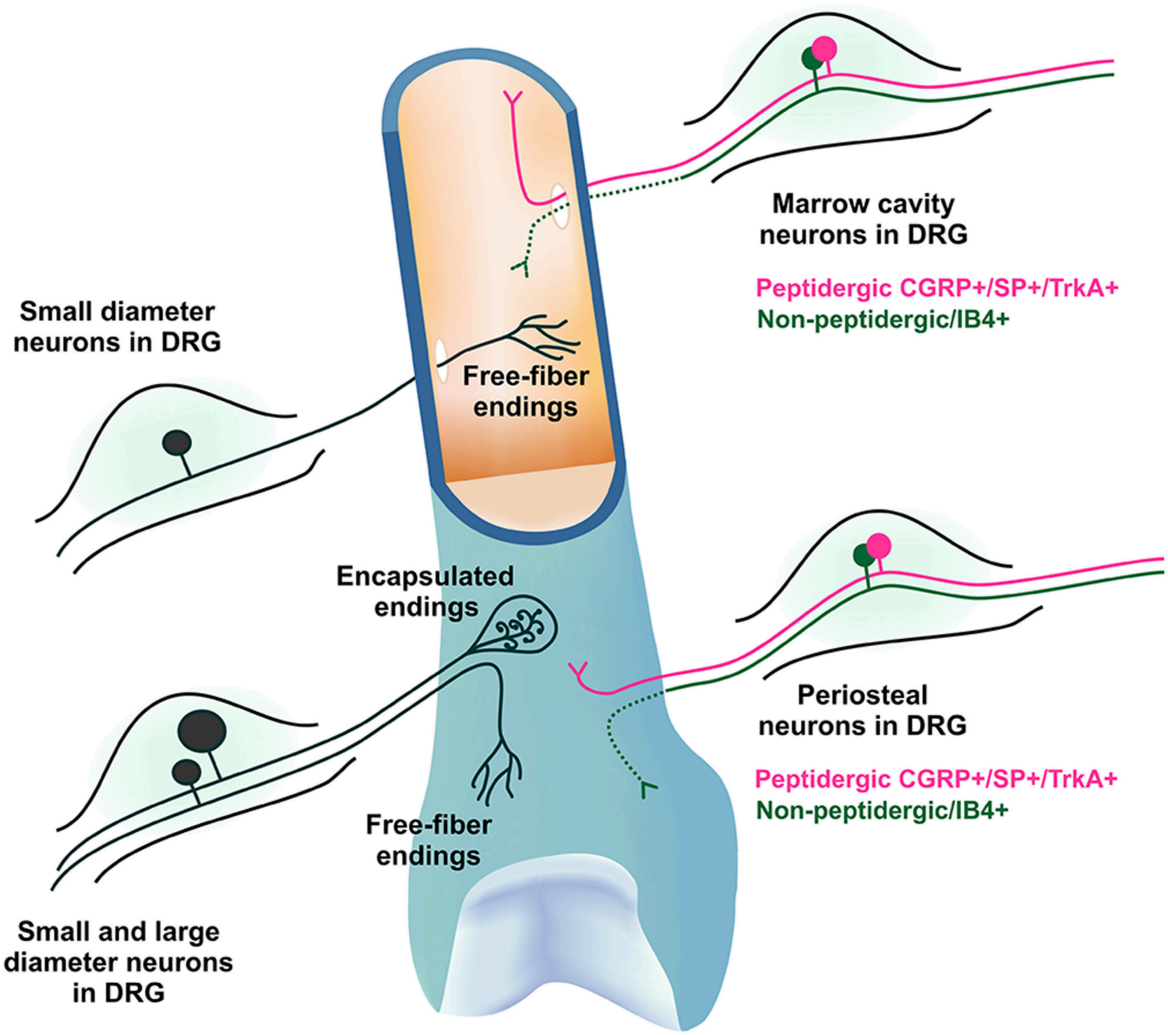
**Morphology and molecular phenotype of sensory neurons that innervate bone**. The DRG soma of primary afferent neurons that innervate the bone marrow and periosteum are mostly small diameter myelinated and unmyelinated neurons with free fiber endings, although some larger neurons with encapsulated endings do exist in the periosteum. They express varying combinations of markers characteristic of nociceptive neurons, including calcitonin gene-related peptide (CGRP), substance P (SP) and the tyrosine receptor kinase A (TrkA), and/or bind isolectin B4 (IB4). IB4 binding has not been observed in peripheral nerve terminals (represented by dotted line).

There are many studies that have reported the existence of primary afferent neurons that innervate bone, and it has become clear that most of these sensory neurons have a morphology and molecular phenotype consistent with a role in nociception (Figure 1). Here we summarize the literature that has contributed to this understanding before discussing in detail the physiology of sensory neurons that innervate bone. For a more detailed review of current literature regarding the morphology and molecular phenotype of sensory neurons that innervate bone, the reader is referred to the following reviews: Mach et al. (2002), Jimenez-Andrade et al. (2010), Mantyh (2014).

Most early studies of the nerve supply to bone documented examples of dissected or silver stained nerve fibers in bone and periosteum but paid little attention to their function (De Castro, 1925; Hurrell, 1937; Takase and Nomura, 1957; Miller and Kasahara, 1963; Cooper et al., 1966; Sakada and Maeda, 1967a; Calvo, 1968; Thurston, 1982). As many of these fibers were in close apposition to blood vessels within the bone, some of the authors suggested an association with vasculature function, but did not comment further. This is somewhat surprising, because damage to bone and associated tissue is clearly associated with pain, suggesting that at least some of the reported fibers in bone must be nociceptors. The use of immuno-histochemical markers for various neuropeptides in more recent reports has provided evidence that nerve fibers innervating mineralized bone, bone marrow, and periosteum are of both sensory and autonomic origin (Duncan and Shim, 1977; Gronblad et al., 1984; Hohmann et al., 1986; Bjurholm et al., 1988; Hill and Elde, 1988, 1991; Mach et al., 2002). The fibers of sensory origin were generally described as having small diameter free fiber endings, although some larger fibers with specialized encapsulated endings have been reported in the mandibular periosteum of cats (Sakada and Maeda, 1967a; Sakada and Aida, 1971b), human long bone periosteum (Ralston et al., 1960) and Haversian canals in canine cortical bone (Cooper et al., 1966). A newly developed technique for selectively labeling peripheral sensory neurons could be useful in confirming a sensory, as opposed to sympathetic origin, for these nerve terminal endings in bone (Kyloh and Spencer, 2014; Spencer et al., 2014).

Nociceptors are generally defined as small diameter thinly myelinated or unmyelinated primary afferent neurons and can be identified by the presence of specific molecular markers expressed on their soma [in the dorsal root ganglion (DRG)] or their peripheral nerve terminals. The DRG soma of primary afferent neurons that innervate the medullary cavity, trabecular bone and the periosteum are almost exclusively small diameter myelinated and unmyelinated neurons that express varying combinations of the markers characteristic of nociceptive neurons, including calcitonin gene-related peptide (CGRP), substance P (SP), and the tyrosine receptor kinase A (TrkA), and/or bind isolectin B4 (IB4) (Gajda et al., 2004; Ivanusic, 2009; Aso et al., 2014). Importantly, these studies have identified sub-populations of sensory neurons that innervate bone on the basis of various combinations of these markers in the rat. For example it is clear that up to half are peptidergic (CGRP+) (Ivanusic, 2009; Aso et al., 2014) and many are non-peptidergic (CGRP- or IB4 binding) (Ivanusic, 2009; Aso et al., 2014), and that approximately two thirds are likely to be nerve growth factor sensitive (TrkA+) whilst others are not (TrkA-)(Aso et al., 2014). It appears that these molecular phenotypes are maintained in peripheral nerve terminals in bone (Mach et al., 2002; Jimenez-Andrade et al., 2010; Castaneda-Corral et al., 2011), although IB4 binding has not been observed at this location (at least in mice; Mach et al., 2002). Whilst there may be some subtle species differences, it is clear that the morphology and molecular phenotype of sensory neurons that innervate tissues within bone are consistent with a role in nociception, and that these features can be used to identify multiple sub-populations of bone afferent neurons (Figure 1). Whether, this molecular heterogeneity is reflected in the physiology of bone afferent neurons remains to be determined. In the rest of this review, we will explore what is known about the physiology of bone afferent neurons.

## Physiology of peripheral bone afferent neurons

The environment in which sensory nerve terminals exist in bone is very different to that in other tissue types. The periosteum lines very hard cortical bone and so sensory nerve endings in the periosteum are easily compressed by relatively low threshold mechanical stimuli compared to endings in more compliant tissue such as skin. Bone marrow is surrounded by non-compliant mineralized bone and contains large populations of progenitor and mature inflammatory cells (and other cell types) that together produce different stimulus conditions in the marrow cavity compared with other tissue. Thus, it is important to consider how primary afferent neurons in each of these different bony compartments respond to noxious stimuli, and how this differs from other tissue types. Here we discuss in detail what is known of the physiology of peripherally located, primary afferent neurons that innervate either the periosteum or the bone marrow.

### Periosteum

There is much greater attention devoted in the literature to periosteal afferent innervation than that of the marrow cavity. This undoubtedly reflects the easier access of the periosteum for experimental assessment than the marrow cavity of bone.

The most detailed series of electrophysiological studies of periosteal innervation was carried out by Sakada and colleagues (Sakada and Maeda, 1967a,b; Sakada and Aida, 1971a,b; Sakada and Onoe, 1971; Sakada and Taguchi, 1971; Sakada and Miyake, 1972; Sakada and Nemoto, 1972; Sakada, 1974; Sakada and Yano, 1978). They made hundreds of recordings from small nerves in an *in vitro* whole-mount preparation of the cat mandibular periosteum describing responses to both noxious and innocuous stimulation of their sensory nerve terminals. Because the receptive fields of periosteal afferents in the preparation were sufficiently discrete, the investigators were able to activate and isolate single units with mechanical stimuli applied at the periosteum. Histology revealed that the cat mandibular periosteum was innervated by small diameter free fiber endings and some larger endings encapsulated by Golgi-Mazzoni corpuscles (Sakada and Maeda, 1967a; Sakada and Aida, 1971b). The free fiber endings were distributed across the entire preparation, whereas the Golgi-Mazzoni corpuscles could only be found at the midline anterior to the mental foramen. Thus, the authors were able to preferentially activate and study free fiber endings by applying stimuli to the periosteum posterior to the mental foramen. They reported that most axons with free fiber endings had small diameters, consistent with a nociceptive function, and systematically explored the response properties of small diameter periosteal free fiber endings in the mandibular periosteum (see below). They also described the behavior of the encapsulated Golgi-Mazzoni corpuscles found anterior to the mental foramen.

Zhao and Levy described a preparation in which they used tungsten wire electrodes to record the activity of trigeminal ganglion neurons with receptive fields on the calvarial periosteum of the rat (Zhao and Levy, 2014). This method allows good isolation of single units, and all of their data are of single unit responses to periosteal stimulation. A total of 115 single units were reported, making it a significant sample population to draw inferences from. They did not comment on the morphology or size of periosteal endings, but they did carefully explore their physiology, predominantly, but not exclusively, in the context of roles in nociception (see below).

Mahns and colleagues used an *in vivo* preparation to explore neurons that innervate the periosteum of the cat humerus (Mahns et al., 2004, 2006). Histology revealed that the small nerve from which recordings were made in this preparation contained only small diameter myelinated and unmyelinated axons (Ivanusic et al., 2006). They were able to selectively activate individual afferent fibers that displayed circumscribed and punctate receptive fields. However, only 15 individual fibers were studied in terms of receptive field characteristics and/or vibro-mechanical sensitivity and responsiveness.

#### Conduction velocities

Conduction velocity is closely related to axon size and can be used to classify primary afferent neurons into a number of functional categories. Afferents with small diameter myelinated (Aδ) or unmyelinated (C) axons and slow conduction velocities are associated predominantly with a nociceptive function (Dixon, 1963; Burgess and Perl, 1973; Lawson and Waddell, 1991; Djouhri and Lawson, 2004; Strassman et al., 2004). C fiber neurons, have the smallest diameter axons (0.6–1.2 μm rat; 1–2 μm cat) and the slowest conduction velocities (<2 m/s rat; <10 m/s cat). Their conduction properties and responses to both heat and chemical stimuli have led to the idea that these are important mediators of slow, burning pain in most tissue systems. The myelinated Aδ fiber neurons have larger sized axons (1.2–4 μm rat; 2–5 μm cat) and faster conduction velocities (2–12 m/s rat; 10–30 m/s cat). Because of their faster conduction, they are believed to be mediators of fast pain. Aβ neurons are also myelinated and have the largest diameter axons (>4 μm rat; >5 μm cat) and fastest conduction velocities (>12 m/s rat; >30 m/s cat). Neurons with large diameter axons, fast conduction velocities and encapsulated endings are typically associated with innocuous (e.g., tactile or kinesthetic) sensibility.

Sakada and colleagues reported that the axons supplying periosteal free fiber endings in cat mandibular periosteum had conduction velocities in the Aδ and C fiber range (2–18 m/s) (Sakada and Maeda, 1967b; Sakada and Taguchi, 1971), suggesting a role in nociception. The axons with encapsulated Golgi-Mazzoni endings had faster conduction velocities (>30m/s) (Sakada and Maeda, 1967b; Sakada and Aida, 1971a), suggesting of a role predominantly in low-threshold mechano-sensibility and not nociception. Zhao and Levy (2014) reported similar distributions of conduction velocity across the Aβ, Aδ, and C fiber ranges in the rat calvarial periosteum, reinforcing the notion that periosteal afferents have roles in both nociception and low-threshold mechano-sensibility. In contrast, Ivanusic and colleagues reported histological findings that the nerve to the cat humerus contained only small diameter myelinated and unmyelinated axons (Ivanusic et al., 2006) and conduction velocities on electrical stimulation of the periosteum that were confined to a range consistent with Aδ and C fiber classification (<30m/s) (Mahns et al., 2006). However, the conduction velocity of only four periosteal afferent fibers was presented, so their sample size is limiting. It is possible that sampling a broader area of the periosteum of the cat humerus may have uncovered units with faster conduction velocities. Alternatively, it might be that units with faster conduction velocities and larger axons are more common in the skull (Sakada and Taguchi, 1971; Zhao and Levy, 2014) compared with the appendicular skeleton (Mahns et al., 2006). Nonetheless, the findings from all investigators indicate that the overwhelming majority of periosteal afferents have conduction velocities consistent with a role in nociception, and likely contribute to sharp, fast (Aδ) or slow burning (C) pain. These types of pain have indeed been reported in humans subjected to periosteal stimulation (Inman and Saunders, 1944), and have been suggested to contribute to pain profiles in a number of animal studies (Martin et al., 2007).

#### Mechanical response properties

All of the above investigators have reported periosteal afferent units to be mechanically sensitive. Sakada and colleagues recorded many hundreds of mechanically sensitive units in their series of papers exploring the cat mandibular periosteum but these studies do not reveal the relative proportion of afferent fibers that were mechanically sensitive because they studied only those that could be identified with mechanical stimuli. In contrast, nearly all of the units (113/115) that could be activated by electrical stimulation of the calvarial periosteum were mechanically sensitive (Zhao and Levy, 2014), suggesting that the overwhelming majority of periosteal afferents are mechanically sensitive in this preparation. Similarly, all 15 of the sensory neurons identified with electrical stimulation of the periosteum of the cat humerus could be activated by mechanical stimuli (Mahns et al., 2006).

The threshold to activation is an important property of sensory neuron physiology that informs how easily a stimulus is transduced at the periphery. The threshold to activation for mechanically sensitive primary afferent neurons is useful in defining their functional classification. For example, most low-threshold mechanically sensitive units have a role in innocuous sensibility, whilst those with high thresholds usually have a role in nociception. Peripheral sensory neurons can also adapt in different ways to the application of a constant mechanical stimulus. For rapidly adapting neurons the discharge frequency declines very quickly and the response to the mechanical stimulus is transient such that impulses only occur at the onset or offset of mechanical stimulation. This provides for clear temporal localization of mechanical stimuli and is characteristic of low-threshold mechano-sensory neurons. The Pacinian corpuscle is an example of a rapidly adapting mechanoreceptor. For slowly adapting neurons, the decline in discharge frequency takes much longer, such that the neuron continues to fire for the duration of the stimulus. The majority of nociceptors are classically defined as having a slowly adapting response to noxious mechanical stimulation, meaning that once activated, a nociceptor will remain activated and provide the CNS with information about the duration of the stimulus.

Sakada and colleagues reported that both the large, encapsulated Golgi-Mazzoni endings, as well as the free fiber endings posterior to the mental foramen, could be classified according to their adaptation responses. Golgi-Mazzoni endings were exclusively rapidly adapting, low-threshold units that responded well to vibration, and are akin to the Pacinian corpuscles or other afferents that mediate innocuous tactile or kinesthetic sensibility (Sakada and Maeda, 1967b; Sakada and Aida, 1971a). In contrast, the free fiber endings they recorded from were either rapidly or slowly adapting, and each of these had different response properties. The impulse patterns to pressure stimulation of slowly adapting free fiber endings varied greatly, however, most showed a sharp increase in activity during the dynamic phase of the pressure stimulus, followed by a period of sustained activity characterized by a gradual increase in inter-spike interval as the receptor adapted to the maintained stimulus (Sakada and Taguchi, 1971; Sakada and Miyake, 1972). With an increase in intensity of mechanical stimulation these slowly adapting free fiber endings displayed an increase in frequency of discharge, at least during the dynamic phase of their response (Sakada and Taguchi, 1971; Sakada and Miyake, 1972). Most of these slowly adapting free fiber endings had axons with conduction velocities in the Aδ neuron range (2–18 m/s) and had relatively high mechanical thresholds (Sakada and Taguchi, 1971), suggesting a role in nociception. These findings are consistent with the findings of Zhao and Levy, who reported that 82% of the mechanosensitive afferents in the calvarial periosteum were slowly adapting and most, but not all had conduction velocities in the Aδ and C fiber range. It is noteworthy that both Sakada and colleagues and Zhao and Levy reported some slowly adapting free fiber endings that responded to innocuous stretch of the digastric muscle and/or conducted in Aβ range, suggesting that some could be innocuous mechanoreceptors rather than nociceptors, but these were relatively few in their preparations.

In the studies of Sakada and colleagues, rapidly adapting free fiber endings were identified by their response to vibratory stimuli (Sakada and Onoe, 1971; Sakada and Taguchi, 1971). Threshold to activation was measured as the minimal voltage, applied to the solenoid of a mechanical stimulator, that was required to elicit a 1:1 pattern of firing (one impulse per cycle of vibration) at 10 cycles per second (Hz). Calibration to real force was not presented so it was not possible to compare mechanical thresholds with other studies, but they were able to discriminate between relatively high and low threshold rapidly adapting free fiber endings within their own studies. Rapidly adapting free fiber endings could follow frequencies of vibration well above 300 cycles per second (Sakada and Onoe, 1971). Approximately half of the rapidly adapting free fiber endings in the periosteum had low thresholds and responded to stretch of the digastric muscle that was not considered noxious because it did not elicit a pain reflex or a jaw opening reflex (Sakada and Taguchi, 1971). This suggested that they were low-threshold mechanoreceptors. The other half had relatively high thresholds and were considered to be nociceptors (Sakada and Taguchi, 1971). All 15 mechanically sensitive fibers reported in Mahns, Ivanusic et al. (2006) displayed rapidly adapting properties, as step indentation of the periosteum, by means of either hand-held probes or servo-controlled mechanical stimuli, elicited responses only in association with the dynamic components of the stimulus. Many of these could be activated with very low forces (as little as 0.5 mN) and conducted in the Aδ and C fiber range. They are likely similar to the rapidly adapting free fiber endings defined as low-threshold mechanoreceptors reported by Sakada and colleagues.

The receptive field of a single neuron defines the area of tissue over which an adequate stimulus can elicit activity and therefore influences the capacity of a sensory neuron to detect the location of a stimulus and discriminate between multiple stimuli. Receptive fields of mechanically sensitive units can vary in size for different types of units and in different tissue systems. Sakada and Taguchi (1971) quantified the size of the receptive field of 434 single units innervating the mandibular periosteum. Most units could be activated at multiple, discrete receptive sites over a large area of the periosteum, typically between 2 and 20 mm^2^. There was little difference in the receptive field size of units that responded to stretch of the digastric muscles and those that did not, but there may have been a very modest tendency for slowly adapting units to have slightly larger receptive fields than rapidly adapting units. In the case of the periosteum of the cat humerus, each unit had a receptive field comprised of a single locus and was usually of an approximately oval configuration which ranged from 2 to 4 mm^2^ (Mahns et al., 2006). In this latter study, individual periosteal afferent units could usually be selectively activated with the use of fine stimulus probes, suggesting that there is a limited overlap of the terminal receptive fields of individual fibers in the periosteum.

Finally, it is also possible that other fibers of lesser, or no mechanical sensitivity, innervate the periosteum, because in regions that appeared insensitive to direct mechanical probing, it was possible to selectively activate individual fibers by applying focal electrical stimuli (Mahns et al., 2006). These had conduction velocities in the C and Aδ range. They could respond to changes in temperature or chemical stimuli instead of mechanical stimulation, or they could be similar to silent nociceptors found in other tissue systems, that are typically insensitive to mechanical stimulation under normal conditions, but become mechanically sensitive following inflammation (Grigg et al., 1986; Schaible and Schmidt, 1988; Schaible, 1996).

#### Chemical sensitivity and inflammatory mediators

Chemical sensitivity and sensitization by inflammatory mediators is typical of polymodal nociceptors, particularly those classified as C fibers. Only a single study has tested the chemical sensitivity of periosteal afferent neurons (Zhao and Levy, 2014). In this study, recordings of sensory neurons that innervate the calvarial periosteum were made before and during application of known algesic substances, including potassium chloride (50–500 mM), capsaicin (10 μM) and protons (low pH). Potassium chloride produced a dose dependent increase in ongoing activity of both Aδ and C fiber periosteal afferent units, but capsaicin and low pH rarely altered ongoing activity, and when it did the response was of low magnitude. However, the sensitivity of periosteal afferent units to mechanical stimuli was clearly altered after application of inflammatory mediators. Local applications of a mixture of histamine, serotonin, bradykinin, and PGE2 led to increased ongoing activity in nearly one third of mechanically sensitive Aδ units and one half of C fiber units, and an increase in the mechanical responsiveness of nearly half of the Aδ fiber units and all of the C fiber units tested. The mechanical sensitization was long lasting (often more than 30 min) and was related to peri-orbital tactile hypersensitivity, commonly linked to primary headache attacks. Thus, sensitization of periosteal afferent neurons can occur and likely contributes to altered pain processing in pathology. In addition to providing evidence that periosteal afferents can be sensitized, these findings also highlight that some periosteal afferents can be activated by multiple stimuli and can therefore be considered polymodal. The idea that periosteal afferent units are polymodal was not explored in any of the other studies of periosteal innervation described above.

#### Response to changes in temperature

Sakada and Nemoto (1972) recorded both multi-unit and single unit responses to dynamic changes in temperature applied to the periosteum. This was done by recording from periosteal nerves whilst cooling the bath solution from 32 to 27°C and then warming back to 31°C. There was no spontaneous activity in the recordings at 32°C. The number of units active in the multi-unit recordings increased as cooling was applied, suggesting progressive recruitment of temperature sensitive periosteal units. The discharge frequency of the whole nerve recordings also increased with cooling, indicating that at least some of these multi-units can code for the intensity or rate of change in temperature. Interestingly, the units that responded to cold became silent as the temperature was changed to one that is warming instead of cooling, suggesting they sense changes in temperature rather than absolute temperature. This is similar to cold receptors in the cornea (Carr et al., 2003). As the warming continued, different units began responding to the warming stimulus, and they too were capable of coding the intensity or rate of change in the warming stimulus. Thus, some periosteal afferents respond to innocuous cooling and some to innocuous warming. Sakada and Nemoto (1972) further explored the temperature sensitivity of 93 mechanically sensitive periosteal units isolated from the whole nerve recordings by applying temperature changes to discrete receptive points of single units on the periosteum. Their responses to cooling were assessed down to at least 17°C (sometimes even down to 0°C) and to warming up to a maximum of 45–50°C, ranges that include temperatures that are considered to be noxious. 20/93 of these did not respond to temperature changes at all, even when these changes were extreme. 24/93 responded to cooling but not warming, and 19/93 responded to warming but not cooling. 30/93 responded to both cooling and heating. Those in the latter three categories were only tested to the point where threshold to activation was reached for either cooling or heating, and so it is not entirely clear if all of these responded into the noxious range of temperatures, although for many the threshold to activation itself occurred at noxious temperatures. It has to be noted, however, that some Aδ mechanically sensitive nociceptors in the skin have very high thresholds to heat (median threshold greater than 53°C) but can become more sensitive to thermal stimulation following sensitization (Type I Aδ nociceptors; Treede et al., 1995), and so the temperatures used in the study of Sakada and Nemoto may not have been sufficient to activate some of the units they reported to be purely mechanically sensitive.

Taken together, these findings suggest that many periosteal free fiber endings are responsive to innocuous and noxious thermal stimuli. However, it is unlikely that physiological changes of temperature around bone are great enough to activate these fibers (Sakada and Nemoto, 1972). It is also unlikely that they are activated directly by pathological changes, because even warming produced by inflammation *in vivo* (Segale, 1919) would not cause a sufficient change in temperature to activate these receptors. It is of course possible that whilst inflammation does not produce changes in temperature that could activate periosteal fibers directly, it can alter their sensitivity to thermal stimuli such that they become more responsive to changes in temperature. Indeed inflammation is known to increase the thermal sensitivity of primary afferent neurons in many other tissue systems (Cervero and Laird, 1999; Ren and Dubner, 1999b). Thus, activation of periosteal afferents by temperature may be possible under highly abnormal or pathological conditions.

#### Summary/conclusions

**Figure 2:**
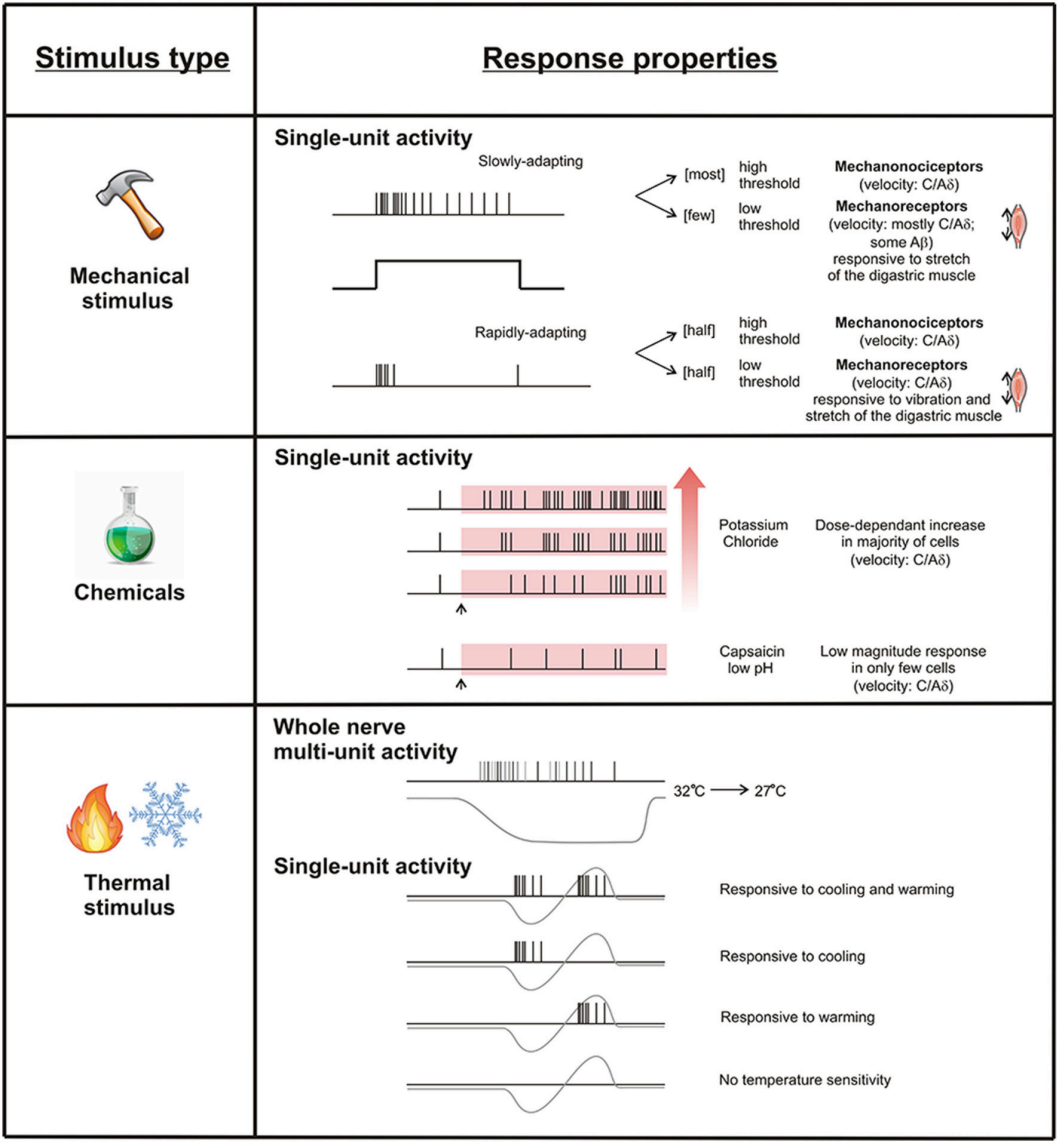
**Response properties of periosteal free fiber endings**. Single periosteal units respond to mechanical, chemical, and thermal stimuli. Mechanically sensitive units can be classified according to their threshold and adaption profile. Potassium chloride activates most periosteal units dose-dependently, but capsaicin and low pH only rarely activates them. Some periosteal units respond to cooling and heating, some to cooling but not heating, some to heating but not cooling, and some to neither.

Figure 2 summarizes what we know about the physiology of sensory neurons that innervate the periosteum. The periosteum is innervated both by large diameter, fast conducting units with encapsulated endings that are likely to provide information about innocuous sensibility and by small diameter, slower conducting units with free fiber endings typical of nociceptors. Activation of the latter is likely to generate the pain experienced during pathology involving the periosteum. Their response properties, including conduction velocities and responses to chemical stimuli suggest roles in both fast, sharp bone pain, and also slow burning bone pain. However, there is evidence that some free fiber endings in the periosteum are activated by relatively low thresholds. In skin, low threshold mechanical stimulation of some small diameter, myelinated (Burgess and Perl, 1967; Burgess et al., 1968; Koltzenburg et al., 1997) and unmyelinated (Vallbo et al., 1993, 1999; Olausson et al., 2002) fibers produces percepts that have been described as non-painful. Whether low threshold free fiber endings have a role in innocuous mechanosensory perception in bone requires further investigation (Rowe et al., 2005), but it seems unlikely because it is difficult to conceive of any stimulus that could be applied to bone that is not considered painful. The alternative is that they may be easily activated by low threshold mechanical stimulation of the periosteum because of its tight relationship with the underlying, hard, bony surface and could therefore contribute to periosteal pain perception. There is also evidence that large diameter neurons in other tissue systems can contribute to pain processing (Djouhri and Lawson, 2004). Thus, it is possible that the reported large diameter encapsulated endings do have some, as yet unidentified role to play in bone pain as well.

### Bone marrow

Brjussowa and Lebedenko (1930; cited in Furusawa, 1970) studied the reaction of dogs during the injection of physiological saline under pressure into the bone marrow cavity. Monitoring blood pressure and respiration, they observed that animals experienced strong pain-like behaviors during the injection. This suggested that there must be sensory nerves in the marrow cavity that responded to increased pressure. Only two published studies however, have investigated the physiology of sensory neurons supplying the marrow cavity of bone (Furusawa, 1970; Seike, 1976). In these studies, whole nerve recordings were made from branches of the tibial nerve whilst mechanical, thermal or chemical stimuli were applied to the marrow cavity. No attempt was made to explore the activity of single units in these studies. On the basis of histological findings, the investigators suggested that recordings were exclusively from Aδ and C fiber units. However, conduction velocities were not confirmed in either study.

#### Mechanical response properties

Mechanical stimuli have been delivered to the marrow cavity by increasing the normal intra-osseous pressure through infusion of isotonic saline into the medullary cavity of the bone. Normal intra-osseous pressure and the extent of the increased pressure were monitored via a manometer attached to the system. In the dog, the normal intra-osseous pressure of the tibial marrow cavity was in the range of 30–50 mmHg and an ~3–5 times increase in intra-osseous pressure (to 100–130 mmHg) was sufficient to mechanically activate multiple units in whole nerve recordings (Seike, 1976). Similar activation thresholds for whole nerve activity had previously been described by Furusawa (1970). These are very high thresholds that are unlikely to be experienced under normal physiological conditions. However, increases in intra-osseous pressure (~3–5 times that of normal intra-osseous pressure) are experienced in pathological conditions such as intra-osseous engorgement syndromes (Lemperg and Arnoldi, 1978; Arnoldi et al., 1980). In these cases, the increase in pressure is associated with pain which can be relieved by fenestration, suggesting that increased pressure in the marrow cavity produces pain.

In the study of Seike (1976) the discharge frequency increased immediately after the start of the pressure stimulation suggesting a short latency response to mechanical stimuli. The rate of discharge generally had a tendency to increase as pressure increased, and when a stable ramp of pressure was applied, the response gradually subsided as receptors appeared to slowly adapt. However, it should be noted that single units were not isolated in these studies, and so it is not clear to what extent this adaptation profile is really true of individual sensory neurons that innervate the marrow cavity.

#### Chemical sensitivity

Only one study has investigated the response of sensory receptors in bone marrow to chemical substances (Seike, 1976). Intramedullary administration of known algesic substances (potassium chloride, acetylcholine, histamine, serotonin, and bradykinin) to the bone marrow cavity produced an increase in whole nerve ongoing activity within a few minutes of injection at concentrations comparable to those reported for activation of muscle nociceptors (Fock and Mense, 1976). Whilst the increase in ongoing activity and the latency of the response were reported to be largely dependent on the concentration used for each substance, the extent of these changes was not quantified.

#### Thermal sensitivity

Seike (1976) attempted to record whole nerve activity in response to changes in temperature within the marrow cavity following a reduction in blood flow produced by ligature of the femoral artery or application of vasoconstrictors. Earlier work had reported that these manipulations produce a decrease in temperature of the bone marrow cavity (Yamada and Yoshino, 1977). The frequency of discharge in the whole nerve recordings increased within 5 min of ligature and then gradually decreased back to control levels over the next 15 min. Whilst there was a small decrease in temperature at 5 min, the change in temperature in the subsequent 15 min did not appear to correlate well with frequency of discharge. Intra-osseous injection of adrenaline and noradrenaline (vasoconstrictors) also increased the whole nerve discharge rate. Both substances produced a more significant fall in temperature within the marrow cavity, and a greater change in rate of discharge of the whole nerve, than that generated by the ligature of the femoral artery. As was the case for ligature of the femoral artery, the relationship between the change in temperature and discharge rate was not clear after the initial period of activation. Although, it seems plausible that the thermal change contributes to increased activity in the whole nerve, hypoxia is also likely to contribute to the response. Arterial occlusion results in severe hypoxia and an increase in inflammatory and/or other chemical mediators (Paterson et al., 1988). Indeed some inflammatory mediators have been shown to change whole nerve activity when applied directly to the bone marrow (see above). Thus, the change in temperature in this study may not have been the stimulus that is actually driving change in activity in the whole nerve reported by Seike (1976).

#### Summary/conclusions

**Figure 3:**
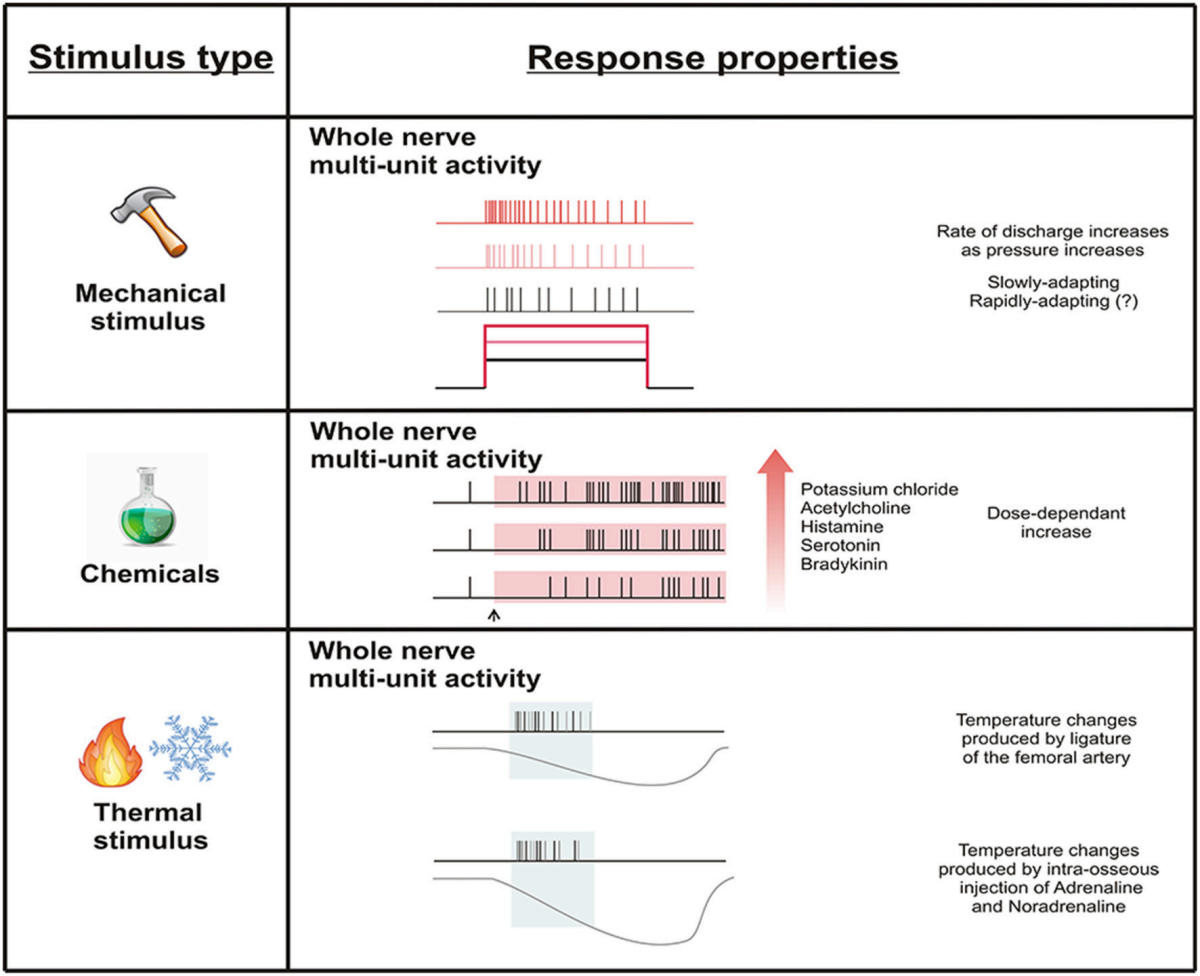
**Whole nerve activity subsequent to mechanical, chemical, and thermal stimulation of the bone marrow**. Whole nerve activity increases in response to mechanical stimulation delivered by increasing intra-osseous pressure. Chemical stimulation dose-dependently increases whole nerve activity. Temperature changes produced by reductions in blood flow appear to influence whole nerve activity, but this could be due to other factors associated with interruption of blood supply to the bone marrow (e.g., ischemia). Single units have not been tested for response to stimulation of the bone marrow.

Figure 3 summarizes what we know about the physiology of sensory neurons that innervate the bone marrow. In contrast to the periosteum, there is little known about the activity of afferent neurons in the bone marrow cavity. Whilst whole nerve activity has been reported subsequent to mechanical, chemical, and possibly thermal stimulation applied to the marrow cavity, the response of single units has not been investigated. Thus, it is not clear if and how single neurons that innervate the marrow cavity respond to mechanical, chemical or thermal stimuli, or if they respond to multiple stimulus types, as is the case for polymodal nociceptors in other tissue systems. It is also unknown if they can be sensitized by inflammatory mediators or other chemical stimuli.

## Physiology of central pathways that code information about bone pain

### Spinal cord

Only a few animal studies have attempted to document the physiology of spinal neurons involved in bone nociception. Most of these have relied on studies of activity dependent Fos expression. Fos is a protein that is produced in the nucleus of cells following expression of an immediate-early gene c-fos (Coggeshall, 2005), and noxious stimuli are known to induce c-fos expression in neurons that possess the gene. The presence of the Fos protein, which can be labeled immunohistochemically, can therefore be used to identify the location of neurons that have been physiologically activated by noxious stimuli. Acute noxious mechanical stimulation of bone, applied by bone drilling and raising tibial intra-osseous pressure, induces an increase in Fos expression in the ipsilateral superficial, but not deep dorsal horn of the spinal cord (Ivanusic, 2008; Williams and Ivanusic, 2008). This same pattern of activity has been observed in studies of Fos expression following acute noxious stimulation of cutaneous tissue (Dai et al., 2001; Jinks et al., 2002) and implies that spinal mechanisms that mediate acute pain of cutaneous and bony origin share some common features. The data implicate the superficial dorsal horn of the spinal cord as a region of interest in studies of acute bone pain, but it is not known if this pattern of Fos expression is different when an inflammatory stimulus is given. Indeed, when inflammatory agents are applied to other tissue systems (such as skin), the pattern shifts such that the deep dorsal horn is most active (Coggeshall, 2005). In animal models of bone cancer-induced pain and skeletal fracture pain, it appears there is increased Fos expression in the deep as well as the superficial dorsal horn, and there is a significant positive correlation between Fos expression and bone destruction (Schwei et al., 1999; Jimenez-Andrade et al., 2007). Interestingly, increased Fos was observed in the superficial dorsal horn in these studies only after normally innocuous stimuli were delivered to the femur by gentle mechanical stimulation (palpation). In the normal animal, noxious cutaneous stimulation is required to induce c-Fos expression in superficial dorsal neurons (Hunt et al., 1987; Abbadie and Besson, 1993; Abbadie et al., 1994; Honore et al., 1995; Doyle and Hunt, 1999). This suggests that sensitization of spinal neurons is occurring in bone cancer-induced and fracture pain.

### Ascending pathways

Williams and Ivanusic (2008) used Fos expression in combination with retrograde tracing to identify the ascending targets of dorsal horn neurons activated by noxious mechanical stimulation delivered by bone drilling. They reported the involvement of the spinoparabrachial pathway, but not the spinothalamic tract or the post-synaptic dorsal column in this model of acute bone nociception. This pattern of activation is different to that observed following acute noxious mechanical stimulation of cutaneous and visceral tissues (Palecek et al., 2003). Spinoparabrachial projection neurons originate predominantly from lamina I of the spinal dorsal horn and project mostly to the contralateral lateral parabrachial nucleus (Kitamura et al., 1993; Gauriau and Bernard, 2002; Almarestani et al., 2007). The lateral parabrachial nucleus connects with several areas of the brain implicated in affective-motivational aspects of nociceptive processing and homeostatic responses to nociceptive stimuli, including the amygdala, nucleus of the solitary tract, ventrolateral medulla, periaqueductal gray, medial thalamus, and hypothalamus (Bianchi et al., 1998; Almarestani et al., 2007). This reinforces connectivity consistent with a strong affective component to bone pain. Whilst Williams and Ivanusic did not provide evidence of the involvement of either the spinothalamic tract or post-synaptic dorsal column pathways in bone nociception, they could not rule out the possibility that these pathways may be involved in animal models characterized by inflammatory or chronic cancer-induced pain. As noted above, these sorts of models are characterized by greater Fos expression in cells of the deep dorsal horn, and are therefore more likely to project through spinothalamic tract or post-synaptic dorsal column pathways, because the majority of cells from these pathways originate in the deep dorsal horn of the rat lumbar spinal cord.

### Cortex

Understanding the physiology of cortical neurons activated by noxious stimuli is important because the cortex is critical to the perception of pain. Only a single study has shown cortical activity related to stimulation of bone afferent neurons (Ivanusic et al., 2009). They showed that sensory information from bone reaches the discriminative areas of the somatosensory cortices by electrically stimulating the nerve to the cat humerus and recording evoked potentials on the surface of the primary (SI) and secondary (SII) somatosensory cortex. Importantly, the nerve to the cat humerus contains only small diameter myelinated and unmyelinated nerve fibers, the size distribution (Ivanusic et al., 2006) and conduction velocities (Mahns et al., 2006) of which are consistent with an Aδ and C fiber classification and therefore a role in nociception. Cortical responses evoked by Aδ stimulation in other tissue types have a relatively short latency (within 50 ms) and are thought to reflect mechanisms associated with fast, sharp pain, whilst cortical responses evoked by C fiber stimulation have a longer latency (50–300 ms) and are thought to reflect mechanisms associated with slow, burning pain (Bromm and Treede, 1984; Willis, 1985). Interestingly, the latency (6–11 ms) to onset of both SI and SII cortical responses on stimulation of the nerve to the cat humerus was consistent with activation of Aδ fibers in the peripheral nerve, and may reflect a mechanism for fast, sharp, and well-localized bone pain, of the sort commonly perceived with periosteal stimulation or in breakthrough pain associated with bone cancers. By increasing the intensity of electrical stimulation, the authors were able to show stronger cortical activation, implying that neurons in SI and SII are able to code for the intensity of stimuli applied to bone. They suggested that small stress fractures are therefore not likely to produce significant pain because the intensity of cortical activity may not be sufficient, whilst large breaks or metastases are likely to produce significant pain. This mechanism of coding for the intensity of noxious stimuli is well-documented in animal studies of the cutaneous system (Kenshalo et al., 1988, 2000; Chudler et al., 1990), and findings of functional imaging studies show that it is also likely to apply to humans (Porro et al., 1998; Coghill et al., 1999). However, the investigators failed to observe long latency cortical responses (50–300 ms) that would be consistent with C fiber activation in the nerve to the cat humerus. Whilst they provided evidence that this may be attributable to inhibition of cortical responsiveness following the initial Aδ response, they could not exclude the possibility that either C fiber projections to SI and SII are too widespread to generate focal evoked potentials of the sort that they could record, or that C fiber input from the nerve to the cat humerus does not reach SI and SII at all. It is also possible that the C fiber input instead projects to other cortical territories, such as the insula, or subcortical areas including the amygdala, nucleus of the solitary tract, ventrolateral medulla, periaqueductal gray, thalamus, and hypothalamus, that have been reported to be important in the affective, emotional aspects of pain.

## Pathophysiological changes in animal models of bone pain

A number of animal models of bony pathology have been developed and are being used to explore pathophysiological and neurochemical changes, in both peripheral and central neurons, that contribute to bone pain. The most common model used is the bone cancer-induced pain model that usually involves inoculation of the rodent femur or tibia with tumor cells (Schwei et al., 1999; Medhurst et al., 2002), but models of bone fracture-induced pain are also common (Freeman et al., 2008; Minville et al., 2008).

Several pro-inflammatory cytokines (IL-1β, TNFα, IL-6, and TGFβ) and inflammatory mediators (CGRP) are increased in the DRG in response to bone cancer and fracture (Kon et al., 2001; Cho et al., 2002; Kang et al., 2005; Rundle et al., 2006; Baamonde et al., 2007; Geis et al., 2010; Fang et al., 2015; Hansen et al., 2016). In animals with bone cancer-induced pain there is also increased DRG expression of several membrane receptors/channels (TRPV1, P2X3, ASIC1a/1b, Nav 1.8, and Nav 1.9) which are known to be involved in the transduction of nociceptive stimuli and/or in the excitability of nociceptors (Nagae et al., 2007; Niiyama et al., 2007; Han et al., 2012; Qiu et al., 2012; Liu et al., 2013; Li et al., 2014). Administration of selective antagonists or antisense oligodeoxynucleotides against some of these channels/receptors attenuate pain-like behaviors in animals with bone cancer pain, further reinforcing a role for these molecules in bone pain (Ghilardi et al., 2005; Gonzalez-Rodriguez et al., 2009; Kaan et al., 2010; Miao et al., 2010). In other tissue systems, inflammatory mediators sensitize peripheral nociceptors, and changes in membrane receptors/channels are likely to be involved (Kidd and Urban, 2001). However, there is no evidence that any of these inflammatory mediators directly activate or sensitize bone nociceptors, or that changes in expression of the various ion channels and receptors alter the physiology or function of bone afferent neurons. Furthermore, the changes observed in the DRG were not localized to sensory neurons that innervate bone; protein expression was assayed using Western blots of whole DRG lysates or quantified by immunohistochemistry performed without retrograde labeling to confirm that DRG neurons innervate bone. Thus, direct evidence for a role of ion channels, receptors, and inflammatory mediators in modulating the activity of peripheral bone afferent neurons, and in regulating pain in bony pathology, is still lacking.

Some direct evidence of sensitization of peripheral nociceptors in bone cancer-induced pain was provided by Cain (Cain et al., 2001) and Uhelski (Uhelski et al., 2013). They reported increased spontaneous activity and reduced heat (but not mechanical) thresholds in peripherally recorded C fiber afferents in animals that had developed behavioral sensitivity in response to injection of tumor cells in and around the calcaneus, but not in control animals. However, in both of these studies, the tumor cells were not clearly confined to the bone, and the C fibers recorded were cutaneous afferents, and so the sensitization was not of bone afferent neurons, but rather of cutaneous afferent neurons innervating the surrounding skin. These studies are more relevant to an understanding of secondary or referred pain associated with bony pathology than the pain perceived on stimulation of the bone itself.

A number of studies have also reported changes in the central nervous system driven by pathology in bone. Increased expression of spinal SP, CGRP, and other inflammatory mediators (TNF, IL-1, IL-6, CCL2, and nerve growth factor) are observed in the spinal cord of rats with fracture-induced and bone cancer-induced pain (Zhao et al., 2013; Shi et al., 2015). Bone cancer induces hypertrophy of astrocytes within the spinal cord, and elevation of the pro-hyperalgesic peptide dynorphin and c-Fos expression in second order neurons of the deep dorsal horn (Schwei et al., 1999; Honore et al., 2000b; Shen et al., 2014). Bone cancer also produces alterations in the physiological response properties of second order neurons in the spinal dorsal horn. In the superficial dorsal horn of animals with bone cancer, there is enhanced spinal synaptic transmission, a higher proportion of wide dynamic range cells, and enlarged receptive field sizes in wide dynamic range cells (Urch et al., 2003; Donovan-Rodriguez et al., 2004; Yanagisawa et al., 2010). Together these changes result in a more excitable spinal cord. They are typical of central sensitization and may underly the development of chronic bone pain.

## Final conclusions

There are many studies that have reported the existence of sensory neurons that innervate the periosteum and marrow cavity, and it has become clear that most of these have a morphology and molecular phenotype consistent with a role in nociception. However, very little is known of the physiology of these neurons. The periosteum has received greater attention relative to the bone marrow, reflecting the easier access of the periosteum for experimental assessment than the marrow cavity of bone. Electrophysiological recordings of sensory neurons in both the periosteum and the bone marrow have confirmed that they both contain nociceptors likely to provide the CNS with information about bone pain. The periosteum (but not the bone marrow) is also innervated by neurons that have properties suggesting they may be stretch receptors or impart innocuous sensibility, although it is not clear if the latter is relevant to stimuli applied to bone. There is only limited evidence that peripheral bone afferent neurons can be sensitized or that they can be activated by multiple stimulus types, and at present this only exists in part for periosteal units. In the central nervous system, it is clear that spinal dorsal horn neurons can be activated by noxious stimuli applied to bone. Some can be sensitized under pathological conditions and may contribute to secondary hyperalgesia or referred pain associated with bony pathology. There are only a few studies of ascending pathways and cortical territories involved. Whilst these provide some clues as to the way information about bone pain is centrally coded, they need to be expanded to further our understanding of other central territories involved. There is a lot more to learn about the physiology of bone afferent neurons, and their central projections, before we approach an understanding that could inform the way we think about and manage bone pain.

## Author contributions

SN and JI both contributed intellectually to the development of this review, including drafting and revising the manuscript. Both approved the final version to be published.

### Conflict of interest statement

The authors declare that the research was conducted in the absence of any commercial or financial relationships that could be construed as a potential conflict of interest.
